# Group 2 ILCs: A way of enhancing immune protection against human helminths?

**DOI:** 10.1111/pim.12450

**Published:** 2017-07-12

**Authors:** N. Nausch, F. Mutapi

**Affiliations:** ^1^ Pediatric Pneumology and Infectious Diseases Group, Department of General Pediatrics, Neonatology and Pediatric Cardiology University Children's Hospital, Heinrich‐Heine‐University Duesseldorf Duesseldorf Germany; ^2^ Institute of Immunology and Infection Research, Centre for Immunity, Infection and Evolution School of Biological Sciences, University of Edinburgh Edinburgh UK

**Keywords:** Group 2 innate lymphoid cells, helminth infection, infection & treatment, innate lymphoid cells, schistosomiasis, T_H_2 immune responses, vaccination

## Abstract

Group 2 innate lymphoid cells (ILC2s) play crucial roles in type 2 immune responses associated with allergic and autoimmune diseases, viral and helminth infections and tissue homoeostasis. Experimental models show that in helminth infections ILC2s provide an early source of type 2 cytokines and therefore are essential for the induction of potentially protective type 2 responses. Much of our knowledge of ILC2s in helminth infections has come from experimental mouse models with very few studies analysing ILC2s in natural human infections. In attempts to harness knowledge from paradigms of the development of protective immunity in human helminth infections for vaccine development, the role of ILC2 cells could be pivotal. So far, potential vaccines against human helminth infections have failed to provide effective protection when evaluated in human studies. In addition to appropriate antigen selection, it is apparent that more detailed knowledge on mechanisms of induction and maintenance of protective immune responses is required. Therefore, there is need to understand how ILC2 cells induce type 2 responses and subsequently support the development of a protective immune response in the context of immunizations. Within this review, we summarize the current knowledge of the biology of ILC2s, discuss the importance of ILC2s in human helminth infections and explore how ILC2 responses could be boosted to efficiently induce protective immunity.

## 1 INTRODUCTION

Group 2 innate lymphoid cells (ILC2s) were originally identified in experimental mouse models of helminth infections. Several studies published in 2010 utilized reporter mouse strains marking either interleukin (IL‐)13^1^ or IL‐4^2^ producing cells to identify a cell type, which did not express classical lineage markers of T, B, NK, myeloid or dendritic cells.^2^ These lineage‐negative innate lymphocytes produced classical T helper type 2 (T_H_2) cytokines in response to IL‐25 and IL‐33. In mice infected with the murine helminth parasite *Nippostrongylus brasiliensis*, these cells acted as an early source of IL‐13 and were essential for timely worm expulsion.^1^, ^2^ These innate cells, later designated as ILC2s,^3^ are now well characterized, and their importance in mediating pathology in asthmatic and allergic diseases as well as in viral infections has been described (reviewed in^4^, ^5^, ^6^, ^7^). Subsequently, further innate lymphoid cells were described mirroring the different adaptive CD4+ T cells; group 1 innate lymphoid cells (ILC1s) are the innate counterparts of T_H_1 CD4+ T cells, ILC2s are the counterpart of T_H_2 CD4+ T cells, and group 3 innate lymphoid cells (ILC3s) mirror T_H_17 and T_H_22 (reviewed in^8^). In contrast to T helper CD4+ T cells, and despite the fact that they are of lymphoid origin, ILCs do not express T‐cell receptors and lack any antigen specificity. The discovery of innate lymphoid cells has introduced a new immunological field and transformed our understanding of innate immune responses and the generation of the adaptive immune system.

Experimental studies have demonstrated that ILC2 cells are involved in tissue repair and homoeostasis^9^ (reviewed in^10^) which is an important consideration for tissue‐dwelling helminth. In addition, the involvement in parasite expulsion in intestinal helminths makes these cells important in immune protection against helminth infection and pathology. In one of only two studies of ILC2s in natural human helminth infection, we have shown that ILC2 cells are diminished in schistosome‐infected children and are restored to levels observed in children who are exposed to infection but remain uninfected following curative antihelminthic treatment.^11^

Within this review, we will discuss the current knowledge of the biology, function and regulation of ILC2s, their “potential” importance in human helminth infections and possibilities of utilizing ILC2 to boost protective immune response induced following treatment and vaccination. This knowledge could inform helminth control efforts as calls for helminth vaccine development escalate in the light of global mandates such as “Sustainable Development Goal 3” advocating for eradication or elimination of helminth infection.

## 2 THE BIOLOGY OF GROUP 2 INNATE LYMPHOID CELLS

In mice, ILC2s were originally identified as a type 2 cytokine expressing cell subset, which could not be classified by conventional lineage markers for T cells, B cells, NK cells, macrophages, dendritic cells, neutrophils, eosinophils, basophils or mast cells, but expressed the common leucocyte antigen (LCA) CD45 and their morphology resembled those of typical lymphocytes.^1^, ^2^, ^12^ Early studies identified the markers IL‐17 receptor B, in combination with IL‐17RA forming the IL‐25 receptor, the IL‐33 receptor (T1/ST2) with varying expression of the stem cell factor c‐kit (CD117).^1^, ^2^ These innate lymphoid‐like cells were given various names including nuocytes,^1^ innate helper type 2 cells (Ih2)^2^ or natural helper cells.^13^ They were enriched in mesentery^13^ and have been shown to express the common gamma chain (γ_c_, CD132)‐associated receptors CD25 (IL‐2Rα) and CD127 (IL‐7Rα). IL‐7 be has been shown to play an essential role in the development and survival of ILC2s and ILC3s.^14^, ^15^, ^16^

**Figure 1:**
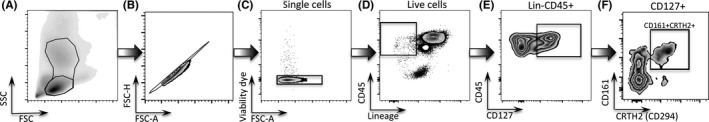
Identification of human ILC2s by flow cytometry as conducted in our studies. PBMCs were isolated from human peripheral blood and analysed by multifluorochrome‐based flow cytometry. PBMCs were gated on leucocytes (A), single cells (B) and live cells using a viability dye (C). Live single cells were gated on lineage negative (CD3, CD14, CD16, CD19, CD20, CD56, CD123, CD11c, αβTCR γδTCR), CD45+ (D), CD127+ (E) and CD161+CRTH2+ cells (F), which finally leads to the identification of lin‐CD45+CD127+CRTH2+CD161+ ILC2s

Human ILC2s were initially described by Mjosberg et al.^17^ as being similar to murine ILC2s in lacking the expression of classical lineage defining markers, but being positive for the leucocyte marker CD45 and the IL‐7Rα (CD127). In addition, human ILC2s express the “chemokine receptor homologous molecule expressed on TH2 cells” (CRTH2=CD294),^17^ a marker well characterized for its expression on human CD4+ T_H_2 cells,^18^ the NK cell receptor NKR‐P1A (CD161)^17^ and ST2^19^ (a member of the IL‐1 family receptors), which is part of the IL‐33 receptor complex.^20^ A combination of these markers is frequently used for identifying human ILC2s as Lin‐CD45+CD127+CRTH2+CD161+(ST2+)^11^, ^17^, ^21^, ^22^, ^23^ as we depict in the flow chart for analysing human ILC2 by flow cytometry (Figure 1).

Apart from the IL‐7Rα, ILC2s express the IL‐2Rα (CD25)^17^ and both IL‐2 and IL‐7 are indispensable for the development, homoeostasis and activation of ILC2s.^13^, ^24^, ^25^ The IL‐7Rα chain forms a heterodimer with the “thymic stromal lymphopoietin” (TSLP) receptor^26^ a further characteristic marker of human ILC2s.^25^ TSLP is able to activate cytokine production by ILC2s, but works more efficiently in combination with IL‐2 and has synergistic effects with IL‐33.^25^ IL‐33 (or IL‐1F11) is a IL‐1 family member and acts via the IL‐33 receptor.^20^ Furthermore, IL‐25 activates cytokines production by ILC2s signalling via the IL‐25 receptor, a heterodimer of IL‐17RB and IL‐17RA. IL‐25, IL‐33 and TSLP can be seen as the classical ILC2 activating cytokines and often referred to as alarmins (alarm signals). Hematopoietic cells can produce alarmins, but the primary sources are nonhematopoietic cells. IL‐33 is primary produced by endothelial and epithelial cells,^27^, ^28^, ^29^ but can be released by macrophages^30^ or dendritic cells.^31^ In contrast, tuft cells, a subset of epithelial cells of the small intestine with previously more or less unknown function, were identified as a major source of IL‐25,^32^, ^33^, ^34^ which is required for ILC2 homoeostasis. The numbers of tuft cells increase significantly when exposed to intestinal parasites.

ILC2s express a variety of additional receptors involved in the activation and homoeostasis. Expression of the IL‐4Rα (CD124) was shown in mice, and basophil‐derived IL‐4 can positively control ILC2s.^35^ As IL‐4 is secreted by ILC2s, IL‐4 could potentially act as an autocrine feedback mechanism for activation of ILC2s. However, the exact role of IL‐4 in controlling activation of human ILC2s is currently unknown. ILC2s are also the main source of IL‐9, another common γ chain (γ_c_) cytokine,^36^, ^37^ with expression of IL‐9 receptor being essential for ILC2 activation, survival of activated ILC2s and finally for efficient helminth worm expulsion in mouse experimental models.^36^ IL‐9 released by lung resident ILC2s plays a central role in the epithelial response to murine *N. brasiliensis* infection by inducing IL‐5 and IL‐13 production.^38^ Gene expression analyses indicated that the IL‐9 receptor is expressed on murine ILC2s, and in humans, expression of this receptor has been shown on blood and lung ILC2s.^21^ The CRTH2 is a crucial marker for the identification of human ILC2s^17^ and for classical T_H_2 cells.^18^, ^39^ The agonist for CRTH2 is prostaglandin (PG)D2, a well‐characterized mediator of allergic asthma^40^ released by activated mast cells. PGD2 is crucial for chemotaxis of T_H_2 cells^41^ and drives accumulation of ILC2s in inflamed tissues.^42^

Murine ILC2s isolated from lymph nodes and the spleen, and to a less extent, ILC2s from the peritoneal or broncho‐alveolar lavage, express major histocompatibility complex class‐II (MHC‐II) molecules. They also express the co‐stimulatory molecules CD80 and CD86.^43^ Expression of MHC‐II in combination with co‐stimulatory molecules allows a direct interaction with CD4+ T cells and can drive CD4+ T‐cell expansion and activation and T_H_2 polarization and is important for efficient worm expulsion in murine infections of *N. brasiliensis*. Accordingly, it had been demonstrated that human ILC2s isolated from peripheral blood express high levels of HLR‐DR, CD80 and CD86.^43^

Similar to all other immune responses, the function of ILC2s needs counter‐regulation allowing control of their function. Type 1 and type 2 interferons can negatively regulate ILC2s,^23^, ^44^ and both types of interferons are long known to inhibit helminth‐driven T_H_2 responses. Additionally, ILC2s can be suppressed by IL‐27^45^ and express the inhibitory receptor killer cell lectin‐like receptor G1 (KLRG1). In human ILC2s, the ligand of KLRG1, E‐cadherin, inhibited expression of GATA3 and production of T_H_2‐cytokines.^46^ GATA3, the transcription factor essential for T_H_2 CD4+ T‐cell polarization and function, is crucial for ILC2 differentiation, maintenance and activation,^24^, ^25^ and also used as identifying marker to distinguish them from other ILC subsets. Furthermore, development, differentiation and function of ILC2s depend on RORα,^16^, ^47^ T‐cell factor‐1 (TCF‐1)^48^ and GFI1.^49^

**Figure 2:**
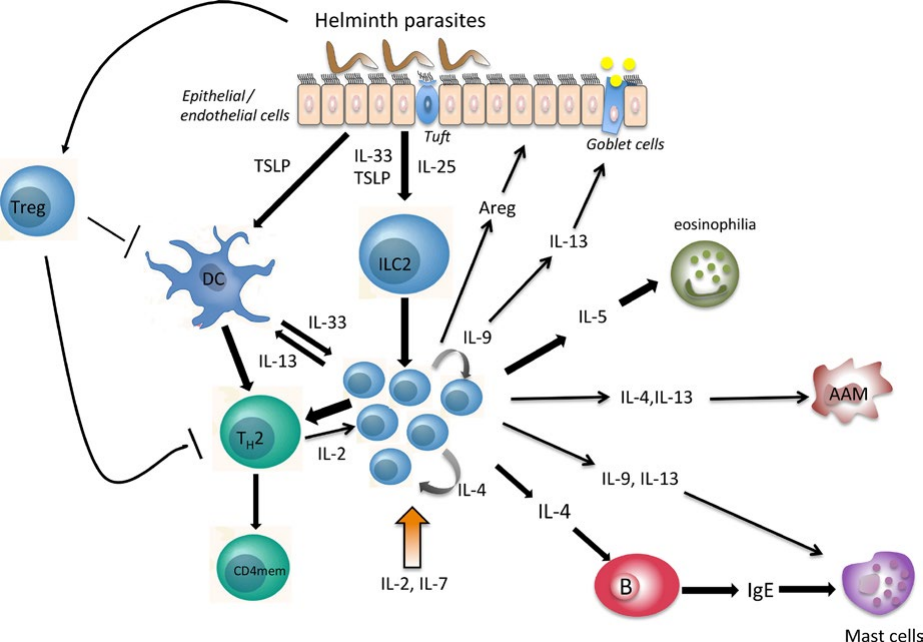
Helminth‐induced immune responses mediated by ILC2s. Helminth parasites trigger the secretion of alarmins by endothelial or epithelial cells (IL‐33, TSLP)^27^, ^28^, ^29^ or by tuft cells (IL‐25).^32^, ^33^, ^34^ Myeloid cells (dendritic cells (DC) or macrophages) can also release IL‐33 and thereby activate ILC2s.^30^, ^31^ ILC2 activation is maintained and multiplied by IL‐4 and IL‐9 (acting in an autocrine manner)^36^ and require IL‐2 and IL‐7 for homoeostasis and activation. ILC2s secrets type 2 cytokines upon activation. IL‐5 induces eosinophilia,^139^, ^151^ and IL‐4 triggers B cells and induces isotype switching to IgE. Furthermore, IL‐13 can activate mucus secretion by goblet cells,^1^, ^16^, ^152^ act on mast cells (potentially in conjunction with IL‐9^152^) and regulate DC migration.^153^ IL‐4 and IL‐13 can also induce alternative activated macrophages (AAM).^154^ ILC2s also secrete amphiregulin (Areg) important for tissue repair.^9^ Furthermore, ILC2s interact with T_H_2 CD4+ T cells (T_H_2), which induces T_H_2 immune response^43^, ^155^ and IL‐2 secreted by T cells could further sustain ILC2 responses and further affect generation of T‐cell memory,^156^ which is altered in chronic helminth infections.^84^ Helminth can induce regulatory T cells (Treg), which potentially can dampen the development of full protective immune response^118^

ILC2s are now considered to play a central role in inducing type 2 immune responses in mice. Following activation, ILC2s secrete type 2 cytokines and activate various and complex immune responses, which are characteristic for type 2 responses including B‐cell activation and isotype switching to IgE, induction of eosinophilia, polarization of alternative activated macrophages and initiation of an adaptive T_H_2 T‐cell response including generation of T_H_2 memory CD4+ T cells as outlined and described in Figure 2.

### 2.1 Common γ_c_ cytokine receptors

Common gamma chain (γ_c_) (CD132) cytokine receptors play a central role in the development, homoeostasis and function of several immune cell lineages and are indispensable for the immune system itself. Therefore, it is not surprising that common γ_c_ cytokines and the corresponding receptors are also essential for the development of ILC2s. The IL‐7Rα chain, forming a heterodimer with the common γ_c_ (also known as common IL‐2 receptor gamma chain), was one of the first surface receptors identified as marker for ILCs, and the development of ILC2s was depending on the common γ_c_ and IL‐7.^13^ The IL‐2Rα is also a marker human ILC2s and provides an important co‐stimulatory signal for the activation of ILC2s.^25^

Using a reporter mouse strain, ILC2s, rather than CD4+ T cells, were also identified as main source of IL‐9 in a model of airway inflammation.^37^ More importantly, IL‐9 acts as feedback signal enhancing the cytokine production by ILC2s. The importance of IL‐9 as a feedback signal was subsequently confirmed in experimental infection with *N*. *brasiliensis*, in which IL‐9 receptor expressing ILC2 is important for restoring tissue damage caused by the lung stage of *N*. *brasiliensis*.^36^, ^38^ Hence, common γ_c_ receptors play a pivotal in the development, maintenance and activation of ILC2s.

In T cells, common γ_c_ signalling is mainly mediated by three pathways: the JAK‐STAT pathway, the mitogen‐activated protein kinases (MAPK)‐Erk pathway and the phosphoinositide 3‐kinase (PI3K) pathway. Binding of cytokines to its corresponding receptors leads to an activation of Janus kinases (JAKs), which are associated with the receptor (reviewed in^50^). JAK activation leads to a phosphorylation of tyrosine residues within the receptor chain causing binding, phosphorylation and dimerization of signal transducer and activator of transcription (STAT), which then translocate to the nucleus and starts specific transcription. There are several STAT molecules partially determining specific effects of cytokines. IL‐2, IL‐7 and IL‐9 mainly activate STAT5, whereas IL‐4 mainly induces the activation of STAT6. Interestingly, the TSLPR, which contains a IL‐7Rα chain, but no common γ_c_, activates STAT5 in a JAK‐independent way.^51^ IL‐2 and TSLP efficiently induce STAT5 phosphorylation in human ILC2s, while IL‐33 elicits a moderate phosphorylation of STAT3.^25^

The JAK‐STAT signalling pathway is tightly regulated to control strength, duration and specificity of activation. Suppressor of cytokine signalling (SOCS) molecule comprises a family of eight members SOCS1‐SOCS7 and the cytokine‐inducible SH2 domain protein (CISH) of which four are shown to be important in T‐cell signalling (CISH, SOCS1‐SOCS3) (reviewed in^52^). The SOCS molecules including CISH have been shown to regulate STAT signalling and modulate T helper polarization.^53^, ^54^, ^55^ Both the MAPK‐Erk and the PI3K pathways play central roles in the development, homoeostasis and functions of several innate and adaptive immune cells. Both pathways contribute to T helper polarization^56^, ^57^, ^58^, ^59^ including differentiation of T_H_2 cells.^60^, ^61^ While the importance of common γ_c_ cytokine receptors for ILC2s is well described for mice, the precise signalling pathways controlling the development and function of human ILC2s remain to be investigated.

## 3 LOCATION OF ILC2s AND THE IMPLICATION FOR HUMAN HELMINTH INFECTIONS

ILC2s have been identified in various tissues. Using reporter mice in experimental models of *N*. *brasiliensis* infection, ILC2s were identified in the spleen, liver, mesenteric lymph nodes, the intestine, fat‐associated lymphoid clusters^1^, ^2^, ^13^ and skin.^62^ In humans, ILC2s have been described in nasal polyps, tonsils, gastrointestinal tract, peripheral blood^17^, ^25^ and the lung.^9^, ^17^ ILC2 are also described in human skin^46^, ^63^ with their migration to the skin being associated with PGD2, the ligand for CRTH2,^63^ and the skin‐homing marker cutaneous lymphocyte antigen.^64^ Overall, mucosa‐associated tissues of the lung, intestine and skin are now widely accepted as the most important locations for ILC2s.

Helminths have complicated and diverse life histories, differing in their route and site of infection, migration within the human host, location of adult worms and exit of juveniles or eggs. This diversity in helminth biology results in heterogeneous acquired immune responses to helminth parasites reflected by fundamental differences in in vitro experiments and in immuno‐epidemiological studies (reviewed in^65^). These life history differences, together with differences in niches relative to the location of ILC2s, imply differences in the encounter between the parasite/parasite products and ILC2 cells. For instance, helminths such as *Schistosoma* spp. (a trematode), *Strongyloides stercoralis* or hookworms (*Ancylostoma duodenale* and *Necator americanus*; nematodes) are skin‐penetrating parasites, meaning that the infective stage and/or the tissue damage caused by the skin penetration can trigger ILC2s.

A percutaneous infection by *Schistosoma mansoni* larvae elicits a transient expression of TSLP and IL‐33.^66^ Although not directly shown, the release of the cytokines is likely to activate ILC2s. Larvae (L3) of vector‐transmitted filarial nematodes (Wuchereria bancrofti *Brugia malayi*,* Loa Loa*,* Mansonella perstans*) also need to penetrate the skin or the bite wound during the blood meal of the vector. *Onchocerca volvulus, Mansonella streptocerca* and *L. Loa* directly develop a cutaneous filariasis with adults residing in subcutaneous tissues. It remains to be investigated whether dermal ILC2s do indeed play a role in initiating antifilarial immune responses following skin penetration and in cutaneous filariasis.

Infection via the skin causes a certain degree of tissue damage^67^ and induces wound healing.^66^ ILC2s are crucial for cutaneous wound healing.^68^ These data suggest that ILC2s may have an additional function in wound healing of damaged tissue caused by skin‐penetrating parasites.

Several helminth species have evolved a critical lung stage, which can be either transient (*Ascaris*,* Schistosoma*,* Strongyloides* spp.) or more persistent (*W. bancrofti*,* B. malayi*,* L. Loa*) (reviewed in^69^). Lung stages of helminths can cause tissue damage in the lung and affect mucosal integrity. In experimental mouse models, the crucial role of ILC2, mediated by IL‐9, acting in autocrine manner, in tissue repair and lung homoeostasis, has been well documented.^36^, ^37^, ^38^ Activated macrophages are also involved in limiting tissue damage during lung migration, a process requiring IL‐4/IL‐13 signalling^70^; although in this former study the exact source of these cytokines was not determined, ILC2s should be considered as source of these cytokines. Mature adults of gastrointestinal helminths (hookworms, *S. stercoralis*) reside in different parts of the intestine and influence and/or damage the epithelial tissue resulting in a release of IL‐25, IL‐33 and TSLP, triggering ILC2s.^71^ As the intestinal tissue is a main compartment where ILC2s are located, it is likely that activated ILC2s play a major role in initiating the immune response in these helminth infections. It will be difficult to prove this role of intestinal ILC2s in a human infection, but mouse experimental studies strongly support this as reviewed in.^72^

Adult schistosomes reside in mesenteric (*S. mansoni*,* S. japonicum*) or in the perivesical venous plexus (*Schistosoma haematobium*)^73^ were they interact with the epithelium. Moreover, eggs released by the females need to penetrate the bladder wall (*S. haematobium*) or migrate to the intestine (*S. mansoni*), damaging epithelial tissue. As outlined above damaging the epithelium could trigger ILC2s, but so far, there are no studies investigating whether schistosomes induce ILC2 directly or indirectly through tissue damage.

ILCs derive from a common lymphoid precursor in bone marrow^74^ expressing the integrin α_4_β_7_, mediating migration to endothelial venues and mucosal tissues, and chemokine receptor CXCR6 mediating migration to the intestine.^75^ Additionally, a lineage‐specific precursor has been identified for ILC2s.^24^, ^47^ However, it has been suggested that ILC2s proliferate within tissues and are rarely replenished from the bone marrow.^45^ More committed progenitors were also identified in secondary lymphoid organs.^76^ Therefore, the contribution of circulating ILC2s from peripheral blood to tissue‐resident ILC2 pool needs to be studied in more detail to allow interpretation of immuno‐epidemiological data based on human blood, as theoretically, blood ILC2s may be important in blood residing pathogens including schistosomes.

Much of our knowledge about ILC2s in helminth infections derives from experimental infection with *N. brasiliensis,* a murine gastrointestinal parasite and most experimental infections cover days or a few weeks following initial infection. In human, however, adult worms can live for years, and in the case of *Schistosoma* spp. even decades.^77^ For people living in endemic areas, an infection does not occur solely at a single time point, but instead occurs more gradually with multiple infection events, resulting in hosts carrying different life stages of the parasites concurrently. Hence, most helminth parasite can cause a chronic long‐lasting disease, which cannot be recapitulated in the mouse experimental model. Hence, knowledge about the function and importance of ILC2 obtained from experimental models cannot necessarily extrapolated to natural human infections.

## 4 ILC2s IN HUMAN HELMINTH INFECTION—WHAT WE DO “*NOT”* KNOW

So far there are very few studies that have analysed ILC2s in natural human helminth infection, a fact, which is not surprising considering the history and biology of ILC2s outlined above. ILC2s constitute a small fraction of human blood leucocytes. In our studies in a Zimbabwean population, a mean of 0.031% (median 0.023%, range 0.003‐0.133, N=72) of live‐gated leucocytes was denoted as ILC2s, a proportion which is comparable to data published elsewhere^17^ and data in Caucasians (unpublished data). Of note, ILC2s are hardly detectable in peripheral blood of naïve mice.^1^, ^2^

In humans, proportions are slightly higher in the skin, ileum, lung and tonsils compared to peripheral blood and are increased in inflamed tissues such as inflamed nasal polyps and skin lesions of patients with atopic dermatitis.^17^, ^46^, ^64^, ^78^ Furthermore, proportions of ILC2s are increased in lung specimens of patients with severe forms of asthma. However, it remains contradictory whether the proportions of ILC2s are higher in lung specimens compared to blood of the same patient.^79^, ^80^ Such analyses of human tissues are informative but are beyond the scope of immuno‐epidemiological approaches such as the ones we have previously published.

Nevertheless, there have been some advances in studying human ILC2 cells in the context of natural human infections. Nutman and colleagues analysed ILCs defined as lin‐CD45+CD127+CD117+ (c‐kit), comprising both ILC2s and ILC3s, in peripheral blood of filarial infected adults (*L. Loa*,* W. bancrofti*,* O. volvulus*).^81^ The frequency of c‐kit+ ILCs and IL‐13 producing cells among c‐kit+ ILCs was increased in filarial infected individuals. The proportion of c‐kit ILCs correlated with IL‐17‐producing CD4+ T cells and ex vivo stimulation of enriched ILCs released IL‐5 and IL‐13, but also IL‐10, IL‐17 and IFNγ.^81^

**Figure 3:**
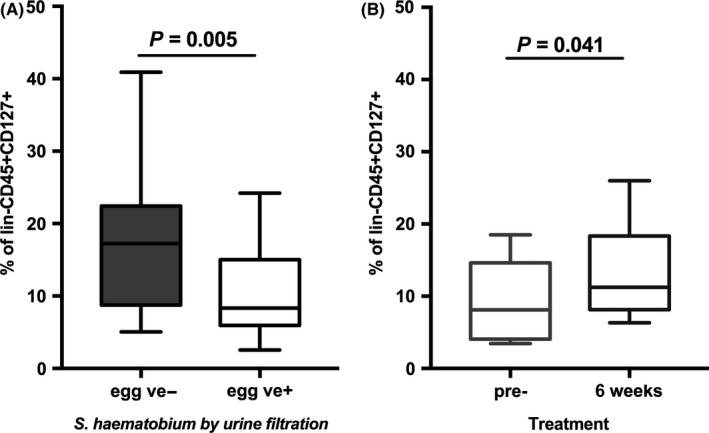
Proportions of ILC2s are diminished in schistosome‐infected children and restored by curative treatment. (A) Proportions of blood CD127+CD294+CD161+ ILC2s were compared between *Schistosoma haematobium* egg‐positive (+ve) children and *S. haematobium* egg‐negative (−ve) children (N=24 per group, age 6‐13 y). (B) Proportions of ILC2s of 12 individuals (aged 6‐13 y) were compared pre‐ vs 6 wk post‐treatment. Individuals were egg positive pretreatment and had cleared *S. haematobium* infections after treatment with the antihelminthic drug praziquantel. Figures are reproduced from data published in^11^

In a different study, we evaluated ILC2s in context of natural infection with *S. haematobium* in Zimbabwean children.^11^ Schistosome‐infected children aged 6‐13 years (as diagnosed by parasite egg excretion) had a significantly lower frequency of ILC2s in the peripheral blood compared to same‐age schistosome‐uninfected children (Figure 3A). In contrast, older infected children (aged 14‐18 years) had comparable levels of ILC2s to uninfected children.^11^ Proportions of ILC2s recovered following curative antihelminthic treatment (Figure 3B). Of note is the difference in these age groups; children are exposed to schistosome infection very young and therefore acquire infection at a young age.^82^ By the time they reach adolescence, they will have experienced several re‐infection events. Thus, it is possible that the ILC2 dynamics are reflecting differences occurring upon first vs repeated infection events. Older egg‐positive children had levels of ILC2s comparable to same‐age egg‐negative children. These older egg‐positive children show a schistosome‐specific antibody profile, which indicates a history of previous infection and also associated with the development of protective immunity, beginning to reduce re‐infection levels.^83^, ^84^, ^85^ This finding may indicate that ILC2s play a more pronounced role in the initiation of early immune response at a stage when effective T_H_2 responses are triggered and a full CD4+ T‐cell‐mediated T_H_2 response has not yet developed. In this context, it would be interesting to perform long‐term follow‐up studies, which analyse whether early changes in the proportion or phenotype of ILC2 are predictive for the development of, or the nature of a protective immune response. The study analysing ILCs in filarial infections mentioned above^81^ found increased proportions of ILCs in infected adults, which may indicate a complete contrasting function of ILCs in adults. For instance, ILC2s could play a role in diminishing tissue damage and promote epithelial healing to limit pathology or alternatively contribute to pathology. Differences between the two studies could reflect differences in the biology of trematodes and filarial nematodes. For instance, filarial parasites frequently harbour *Wolbachia* spp. endosymbionts,^86^ which could trigger TLR responses and potentially modify the response elicited by various ILC subsets including ILC2s and ILC3s. Whereas the precise pattern of TLR expression on ILC2s has not yet been specified, LTi‐like group 3 ILCs have been shown to express TLRs.^87^ Of note, the study by Boyd et al. analysed CD127+CD117+ ILCs comprising both ILC2s and ILC3s.^81^ To analyse the contribution of *Wolbachia* endosymbionts to ILC‐mediated immune responses, interventional studies with doxycycline, which targets *Wolbachia* spp. in filarial infections could be utilized.^88^, ^89^, ^90^ Furthermore, differences in the life cycle and age dynamics in different types of helminth infections could contribute to differences in ILC2s. Therefore, additional observational and interventional studies are required to decipher the precise role of ILC2s in various helminth infections and in the context of the complex dynamics of human helminth infections, which cannot completely be mimicked by experimental models. The mechanism responsible for differences in the frequency of ILC2 in peripheral blood in these human helminth studies remains unknown. One possibility is that one or all of proliferation, survival and homoeostasis of ILC2 is altered during helminth infection. Common γ_c_ cytokine signalling (as outlined above) could be altered during helminth infections. Chronic down‐modulation of IL‐7Rα on memory T cells has been shown for chronic viral infections,^91^, ^92^ a mechanism potentially affecting ILC2s in chronic parasite infections. However, expression of the IL‐7Rα chain on the surface of ILC2 was not altered during schistosome infections.^11^ Regulation of IL‐7 signalling is much more complex and could depend on the availability of IL‐7 and levels of soluble IL‐7Rα (generated by alternative splicing^93^), which has been shown to inhibit IL‐7 uptake^94^ or function as IL‐7 reservoir.^95^ Aberrant levels of plasma IL‐7 and soluble IL‐7R were recently shown in the context of human tuberculosis.^96^ In addition, modulation of downstream signalling in particular of the JAK‐STAT pathway including modulation of SOCS may influence homoeostasis and responsiveness of human ILC2s. Modulation of this signalling pathway in T cells has been shown for various infectious diseases including tuberculosis.^97^ Whether and to which degree common γ_c_ signalling is modulated in ILC2s in particular during helminth infection remains elusive. Furthermore, modulation of signalling via the IL‐9R and TSLPR could be regulated and may provide molecular targets for chemoprophylaxis or therapy.

The impact of nutrition, particularly micronutrients on the immune system, is well established in experimental models. Micronutrient deficiency is widespread in helminth‐endemic areas with vitamin A deficiency being one of the most common. Interestingly, work in experimental studies indicates that vitamin A deficiency is characterized by an increase in ILC2 cells and increased production of IL‐13 by these cells to maintain mucosal barrier immunity to helminth infection under malnutrition.^98^ In addition, recent work has also highlighted that ILC2 cells predominantly depend on fatty acid (FA) metabolism during helminth infection.^99^ The vast majority of the world's malnourished people live in developing countries, where 13.5% of the population is undernourished^100^ and areas of malnutrition largely overlap with helminth‐endemic areas. Therefore, it is important to understand the development and function of ILC2 cells in populations exposed to helminth infection. There are many potential sources of heterogeneity, not least the gut microbiome structure**.** Mouse experimental studies have demonstrated that infection with the helminth *Trichuris muris* significantly altered the host gut microbiome structure, reducing the diversity and abundance of the *Bacteroidetes*,* Prevotella* and *Parabacteroidetes*.^101^ This dysbiosis was associated with a significant reduction in amounts of Vitamin D derivatives and a reduction in the breakdown of dietary plant‐derived carbohydrates involved in amino acid synthesis, with an associated reduction in the weight of the infected animals. We have demonstrated that the gut microbiome structure in children infected with schistosomes differed significantly from that of uninfected children from the same community.^102^ Although the development of ILC2 cells does not seem dependent on the gut microbiome, their function is dependent on the colonization of the gut by commensals (reviewed in^103^). However, the precise mechanisms of how/which signals from the gut microbes interact with the ILC2 to facilitate their maturation and function remains unknown. Answers to these questions will only come from studies conducted in context, in the relevant human populations.

To date, both experimental and human studies have mainly focused on infection. However, there is also another aspect of human helminthiases in which the immune response plays a central role, that is immunopathology. Eggs are mainly responsible for the pathology‐associated schistosomiasis, and egg‐induced immunopathology can occur in the chronic form of the disease. Eggs laid by adults worms, which reside in the vesical plexus of the bladder or mesenteric veins of the liver, can be carried to portal venules in the liver and to the bladder or genital tract where eggs become trapped and eventually form granulomas and can induce immune‐mediated fibrosis. The degree of pathology depends on the balance of type 1, type 2 and type 17 immune responses (reviewed in^104^, ^105^). Severe forms of pathology are associated with T_H_1/T_H_17, whereas mild pathology is associated with a combination of regulatory and T_H_2 response. However, the type 2 cytokine IL‐13 also contributes to hepatic fibrosis.^106^ In mouse models, it has been shown that ILC2s are a likely source for IL‐13 in hepatic fibrosis.^107^ Hepatic IL‐33 triggered the expansion and activation of liver‐resident ILC2s, which produced IL‐13 and mediated fibrosis.^107^, ^108^ In human intestinal schistosomiasis, the majority of patients develop a less severe form of the disease, but about 5%‐10% suffer from hepatosplenic schistosomiasis with progressive fibrosis.^109^ To what extent, hepatic ILC2s contribute to the development of severe forms of schistosomiasis remains to be investigated. Furthermore, the impact of environmental enteropathy, which affects gut permeability, exacerbated by helminth infections has yet to be investigated.^110^

## 5 THE POTENTIAL IMPACT FOR TREATMENT STRATEGIES AND SUCCESSFUL VACCINATION

The development of successful vaccinations against human parasitic infections and in particular against helminth infections has proven challenging. Although there are some promising vaccine candidates for instance, a vaccine against hookworms,^111^ currently there is no licenced vaccine against helminth infections for use in human. The reasons for lack of progress in human helminth vaccinology are manifold. Most helminths have complex life cycles with intermediates hosts and reservoirs and several life cycle stages even within the human host leading to highly variable and complex antigen pattern. Helminths typically induce a type 2 response, which is potentially protective. However, work over the last decade has shown that helminths have evolved immune evasion mechanisms allowing the establishment of long‐lasting infections and modulation of pathology (reviewed in^112^, ^113^, ^114^, ^115^). To do so, helminths utilize immunosuppressive and immunoevasive mechanisms, mediated through various mechanisms. For instance, the importance of regulatory T cells has been shown for filarial^116^, ^117^ and schistosome infections^118^, ^119^ and excretory‐secretory products released by helminth parasites can directly induce regulatory T cells.^120^ In the cases of schistosomiasis suppression of immune responses induced by worms can delay the development of protective immunity.^121^ Mechanisms of how the host eventually manages to express a resistance phenotype have been a subject of our research, leading to the description of the threshold hypothesis^122^; that is, the host needs to experience a threshold of antigens to mount an effective immune response and that these antigens become available following worm death. We and others have also demonstrated the requirement of the ratio of regulatory vs effector cellular immune to favour effector responses for expression of resistance.^118^ However, the precise mechanism of the induction of a protective response remains elusive. The description of the ILC2 cells bridging the innate and adaptive immune system could potentially shed light to this aspect of schistosome immunobiology.

Therefore, apart from the search of new vaccine candidates, new strategies to trigger and boost the development of effective immune responses should be investigated. ILC2s are of major importance for the induction of effective type 2 immune responses (Figure 2) and are in particularly crucial in early immune response and hence are a promising target to boost responses. Here, it is interesting to note that in an experimental mouse model excretory‐secretory products of *Heligmosomoides polygyrus* inhibited the production of T_H_2 cytokines by ILC2s through the blockade of IL‐33,^123^ indirectly indicating the importance for dampening ILC2 responses for parasite survival. Overcoming such inhibition and efficient triggering response mediated by ILC2s could be an important step in triggering protective responses against helminth infections. However, our knowledge of the role of ILC2 biology in helminth infections is still very limited to allow a predication if modulation of ILC2s can improve immune response and thereby improve vaccine efficacy.

For schistosomiasis, protective immune responses can build up over time under constant exposure^124^ and repeated treatment can boost specific immune responses.^125^ Therefore, “Infection and treatment” (I&T) strategies are a potential alternative to induce protective immune responses,^126^ which so far has proven to be the most efficient method to induce protection. The efficacy of this approach has been recently shown for human malaria infections.^127^ Understanding the dynamics of ILC2 involvement in inducing protective immune responses might better inform targeting of treatment. For example, we are currently testing the potential for inducing protective immune responses in schistosomiasis following treatment of the first very infection event. Human immunology and mouse experimental studies of helminths and *Plasmodium* infections suggest that the number of antiparasite treatments required to induce protective immune responses can be reduced by treating people following first infection.^128^, ^129^, ^130^, ^131^, ^132^

## 6 MODULATING ILC2 RESPONSES

Common gamma γ_c_ cytokines and their receptors are crucial for homoeostasis and activation of ILC2s and therefore are potential targets to boost ILC2 responses thereby potentially increase the effectiveness of vaccinations or I&T approaches. IL‐2 therapy has a long history in antitumour therapy,^133^ and therapies with low‐dose IL‐2 are currently tested in autoimmune disease such as hepatitis C virus‐related vasculitis^134^ and type 1 diabetes.^135^, ^136^ Early on, it has been recognized that IL‐2 therapy can lead to increased plasma levels of IL‐5 and eosinophila^137^, ^138^ an effect that, at least in mouse models, is caused by an activation of ILC2s.^139^ Side effects of low doses of IL‐2 are considered to be relatively safe, but in the context of autoimmune diseases are used to expand regulatory T cells (reviewed in^140^), which may contradict attempts to trigger a protective response in helminth infections. However, with detailed investigations of treatment regimes regarding the dose and duration of the IL‐2 therapy might help to tackle this problem. For instance, regulatory T cells may expand only after a few weeks of IL‐2 therapy, whereas ILC2 activation may occur quicker in particular if incorporated in I&T approaches or if applied with vaccinations.

IL‐7, another common gamma γ_c_ cytokine, is also considered for use in cancer^141^, ^142^, ^143^ and chronic viral infections,^144^ highlighting the potential in immunotherapies. This is particularly important in the carinogenic trematodes, *S. haematobium, Opisthorchis viverrini* and *Clonorchis sinensis* where one of the pathological manifestations of these infections is cancer in different organs (bladder, bile duct and liver)^145^ for which we currently do not have any therapeutic interventions beyond surgery. As IL‐7 is crucial for the development and homoeostasis of human ILC2s, its potential to increase responses mediated by ILC2s in vaccination and/or I&T protocols should be investigated.

Apart from the direct use of cytokines in immunotherapies, molecules crucial for the downstream signalling induced by these cytokine could be targeted. Interestingly, the effects by IL‐7 in the study on chronic viral infections were partially mediated by repression of SOCS3.^144^ Hence, targeting the JAK/STAT or the MAPK/Erk pathway including SOCS inhibitors may have the potential to increase ILC2 activation, but also T_H_2 responses in general.^53^, ^146^

The main trigger of the ILC2 activity are the alarmins IL‐25, IL‐33 and TSLP, but their potential as activators in immunotherapy has not been investigated in detail. However, blocking alarmins has been considered for treating allergic diseases,^147^, ^148^, ^149^ but has not really gone beyond experimental testing with only initial studies in human.^150^ ILC2 targeting alarmins could be also used in combination with common γ_c_ cytokines. Overall, specific modulation of ILC2 activity to improve vaccine or I&T‐induced protective immune responses is an exciting idea. Precise treatment strategies need to be carefully approved to avoid induction of regulatory T cells or to avoid the induction of allergic immune responses. Before attempting ILC2 targeting strategies to build up protective immune responses, much more work needs to be done on dissecting mechanisms and signalling pathways in ILC2s.

## 7 CONCLUSIONS

Within a few years of their discovery, ILCs have revolutionized immunology research, added a new layer of complexity to the immune system as a whole and transformed our understanding of how immune responses are initiated and maintained. ILCs have been shown to be important in allergic disorders, autoimmune diseases, viral infections and even in tumour immunology. Experimental mouse models of helminth infections have led to increased understanding of the ILC2 biology and provided mechanistic details of the crucial role of ILC2s in inducing T_H_2 responses. Human studies testing the hypotheses from these mouse models lag behind, creating a knowledge gap. However, the limited studies of ILC2s in the context of natural human infections have already started to yield interesting results on the nature and function of ILC2s. Given the complexity and diversity of human helminth infections, much more work needs to be done to obtain a complete figure about the role of ILC2 and the underlying immunological pathways and mechanisms of their function in human helminth infections. While the study of the role and function of human ILC is still in its infancy, rapid incorporation of the knowledge of these cells in our paradigm of the nature and development of protective immunity is essential for helminth vaccinology and optimal treatment strategies.

## CONFLICT OF INTEREST

The authors have declared that no competing interests exist.
